# Embolic agents in emergency care: a large retrospective cohort study

**DOI:** 10.1186/s42155-026-00651-5

**Published:** 2026-02-03

**Authors:** Manuel Gargiulo, Cécile Di-Rocco, Axel Bartoli, Paul Habert, Jérôme Soussan, Pierre-Antoine Barral, Alexis Jacquier, Vincent Vidal, Farouk Tradi

**Affiliations:** 1 Aix Marseille Univ, LIIE, UR4264, Marseille, France; 2 Aix Marseille Univ, CNRS, CERIMED, UAR2012, Marseille, France; 3 Interventional Radiology Section, Imaging Department, Hôpital La Timone, AP-HM, Marseille, France; 4 Imaging Department, Hôpital Nord, AP-HM, Marseille, France; 5 UMR 7339, CNRS, CRMBM‑CEMEREM (Centre de Résonance Magnétique Biologique et Médicale—Centre d’Exploration Métaboliques par Résonance Magnétique), Aix-Marseille Université, Marseille, France

**Keywords:** Interventional radiology, Emergencies, Embolization, Therapeutic, Hemorrhage

## Abstract

**Rationale and objectives:**

Emergency embolization is a cornerstone in the management of active bleeding and vascular lesions at risk of bleeding. This study aims to provide a comprehensive overview of emergency embolization technical modalities in a large cohort of patients, with a focus on embolic agent selection depending on vessel and lesion characteristics.

**Materials and methods:**

This retrospective study included consecutive patients who underwent emergency embolization procedures in an interventional radiology department between 2022 and 2024. Data collection included precise type specifications and quantity of embolic agents, diameter and location of targeted vessel, vascular-type lesions, etiology, and technical success rate.

**Results:**

A total of 304 patients (320 procedures, 465 artery embolizations) were analyzed. Most target arteries measured 1.0–1.9 mm in diameter (42.6%). For arteries < 1 mm (13.8%), liquid agents were preferred (46.7%), whereas coils were the most widely used in larger vessels. In active bleeding (32.7%), liquid agents (38.6%) and coils (37.3%) were the most commonly used. For spontaneously resolved bleeding lesions and pseudoaneurysms (33.1% and 14.2% of lesions, respectively), coils were widely used (34.9% and 50.8%, respectively). Coil use was associated with the highest procedural cost. Technical success was achieved in 99.1% of procedures.

**Conclusion:**

In emergency embolization, small-caliber vessels (1.0–1.9 mm) were the most frequently targeted. In cases of active bleeding, liquid embolic agents were favored, with high technical success. Notably, a substantial proportion of lesions were identified on CT without ongoing bleeding at the time of embolization.

**Level of evidence:**

Level 4, Case Series.

**Supplementary Information:**

The online version contains supplementary material available at 10.1186/s42155-026-00651-5.

## Introduction

Emergency embolization has become crucial in the management of active bleeding or vascular lesions at risk of bleeding, whether traumatic or not. It can prevent or rapidly control bleeding through targeted occlusion of pathological blood vessels using a variety of embolic agents [1]. Over the years, emergency embolization has undergone considerable development, and in many centers has become a first-line treatment option for patients who are stable or stabilized after intensive care [2, 3]. While it has an obvious role in traumatic contexts, it is increasingly requested in the decompensation of chronic pathologies, such as hemoptysis and local vascular tumor invasion, as well as in the management of postoperative, post-interventional, and drug-related iatrogenic complications [4, 5].

These procedures require specific interventional radiology training, with in-depth knowledge of vascular anatomy, as well as expertise in available embolic agents, and appropriate techniques for the different anatomical locations and types of vascular lesions. Indeed, the choice of embolic agent is a determining factor in the success of the procedure and depends on several factors, such as the type of vascular lesion, the size, location, and blood flow of the target vessel, and the degree of emergency [6]. The use of resorbable gelatin in the context of delivery hemorrhage or retroperitoneal bleeding, as well as metallic agents such as coils, plugs, and stents, is now well known and mastered by radiologists trained in embolization [7]. However, the advent of liquid agents, such as cyanoacrylate adhesives or ethylene vinyl alcohol (EVOH) copolymer-based agents, seems to have changed practices [8, 9]. Few studies have compared the cost of liquid agents with other agents, but they seem to indicate that they could be financially economical [10, 11]. Also, publication bias means that few reports mention procedures with abstention or technical failure, and their proportion is unclear.

The rapidity and efficacy of vascular occlusion are paramount in emergency settings, and unfamiliarity with certain therapeutic options or agent characteristics can lead to delayed treatment, with longer decision-making times, initially ineffective embolization, or even procedural failure. The aim of this retrospective study is therefore to reinforce our knowledge of current emergency embolization practice by analyzing the embolic materials used according to angiographic findings.

## Materials and methods

### Study population

This observational retrospective unicentric study received approval from the Local Ethics Committee (No. PADS25-103). Informed consent was waived because of the study's retrospective nature, and the analysis used anonymized clinical data. All patients who underwent emergency peripheral—neither neurological, nor cervico-facial, nor obstetrical—embolization procedures in our center between 2022 and 2024 were consecutively selected. Non-arterial embolization procedures and cases with incomplete data were not included. The study adhered to the recommendations outlined in the STROBE (Strengthening the Reporting of Observational Studies in Epidemiology) guidelines [12].

Emergency embolizations corresponded to all embolization procedures performed without prior scheduling. For all patients, the therapeutic strategy was decided in a multidisciplinary discussion between emergency and intensive care physicians, diagnostic and interventional radiologists, and surgeons, in which CT imaging plays a central role for both diagnosis and triage. Patient’s informed consent has been obtained, if possible given the emergency context, after clear, objective, and intelligible information had been provided by the interventional radiologist. All procedures were performed by senior interventional radiologists under fluoroscopic guidance, using a standardized departmental protocol involving right common femoral artery access under ultrasound guidance. The first-line approach consisted of a 4-Fr coaxial access system with a microcatheter. Escalation to larger introducer and catheter (5-Fr), use of triaxial technique, and the choice of microcatheter were not protocolized and were left to the operator’s discretion according to personal practice.

### Data collected

The location and type of underlying vascular abnormality were recorded, with the latter classified as follows: active bleeding, resolved bleeding (locoregional hematoma, without any of the other lesions listed visible on angiography), aneurysm, pseudoaneurysm, arterial hypertrophy (bleeding due to pressure differential, mainly in cases of hemoptysis), arteriovenous fistula, diffuse microvascular lesion (found in traumatic spleens with diffuse petechiae), venous or arteriovenous malformations.

Procedure sheets prospectively collected data on the devices used. Reports filled in at the end of the procedure attested to medical indication, technical success (i.e., no residual lesions of the targeted vessel in digital subtraction angiography), and potential complications.

Data extraction was performed using Xplore Exploitation Radiology Information System software (version 7.2.35.16; EDL). A comprehensive retrospective review of clinical, imaging, and procedure material data was performed by a junior interventional radiology fellow with 2 years of experience. All uncertain or ambiguous cases were jointly reviewed with a senior interventional radiologist with 9 years of experience.

Angiographic data and measurements were performed on Universal Viewer (version 7.0; General Electric Healthcare). Clinical data included the time and context of occurrence. No clinical follow-up data were included. Imaging data extracted from angiography included diameter, location, and lesion type of the artery involved, technical success (same definition as above, i.e., no residual lesions of the targeted vessel in digital subtraction angiography), and intra-procedural complication assessment. Complications were classified according to the 2017 Cardiovascular and Interventional Radiological Society of Europe (CIRSE) complication classification system [13]. Procedure material data detailed the precise type specifications, quantity, and price of embolic agents chosen at the discretion of the operator. The unit price is extracted from the database of the 452 embolization cases in the study. Coil detachment handles, as well as the use of lipiodol for cyanoacrylate, were included in the analysis. There was no standardized protocol for the selection of embolization material. Exact cost was obtained from the list of products and services database of the authors' country. The cost by embolization according to the type of agent was calculated by multiplying the unit price of the agent by the number of units used per embolization, to which the price of any accessory devices was added—like release handles. “Post-interventional” included complications following surgery, interventional procedures such as biopsies or drainage, or technical medical acts. “Diffuse microvascular lesions” corresponded to traumatic spleens with diffuse petechiae.

### Statistical analysis

Data were analyzed using descriptive statistical measures, including number and percentage for categorical variables, and median (Q1–Q3) or mean ± standard deviation for continuous variables. Analyses were performed using Excel (version 16.41; Microsoft Corporation) and R software (version 4.4.2). Data generated or analyzed during the study are available from the corresponding author upon request.

## Results

Some patients required more than one embolization session during the study period, either for persistent or recurrent bleeding.

**Fig. 1:**
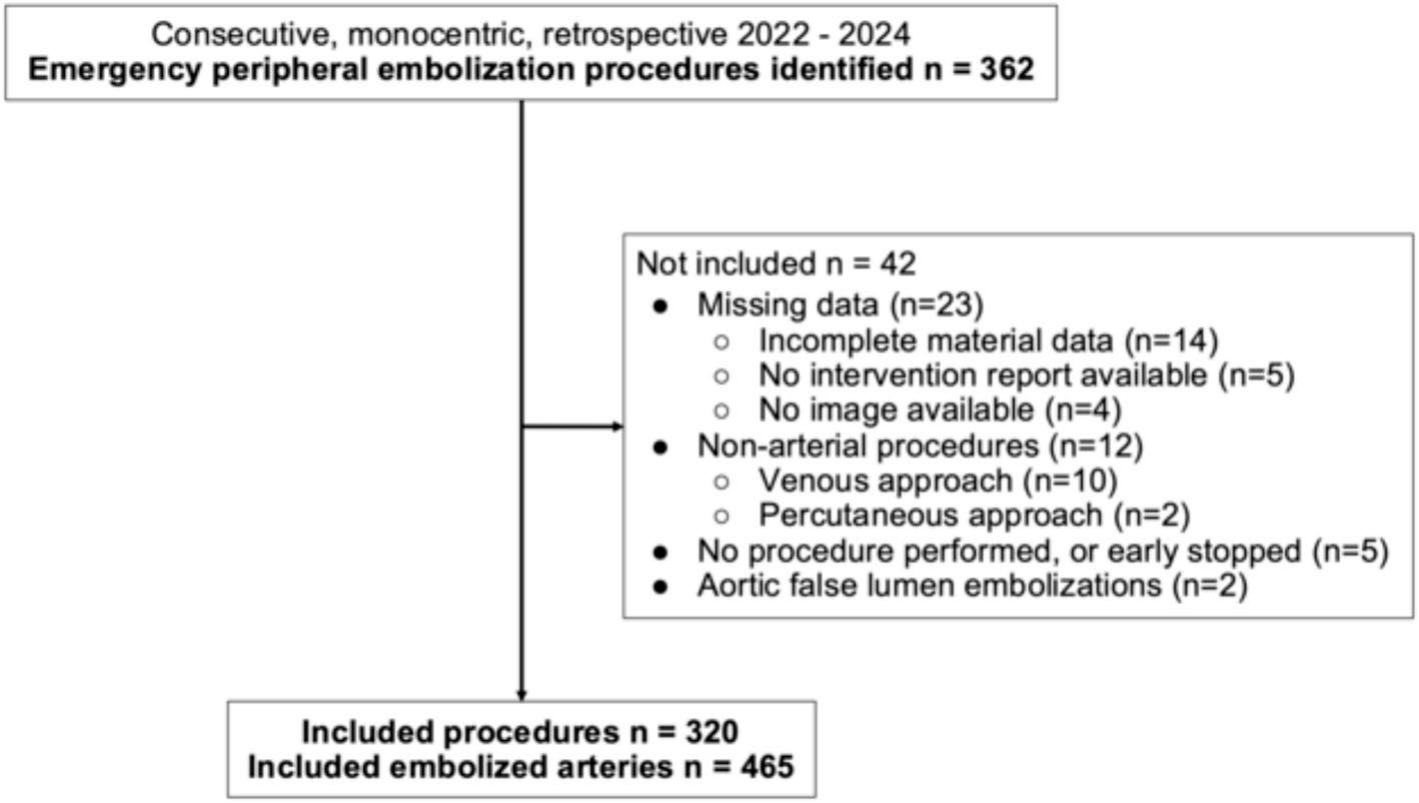
Flow diagram for Inclusion

This single-center retrospective study included 304 patients who underwent 320 procedures between 2022 and 2024, during which 465 target arteries were embolized (Fig. 1). Patients who underwent more than one embolization session were treated during separate hospitalizations, with no repeat procedures performed during the same hospital stay. Vascular lesion characteristics are presented in Table 1. They mainly occurred in iatrogenic (*n* = 194 of 465, 41.8%) and traumatic (*n* = 140, 30.1%) contexts, whereas oncological contexts account for only 9% (*n* = 42) of cases. In our cohort, 46 (9.9%) embolizations were realised on patients who had presented hemodynamic instability, including 12 cases involving muscular locations (10.9% of instability cases).

**Table 1:** Arterial lesion characteristics

Characteristic	Number (%); *n* = 465	Median (Q1–Q3)	Missing data *n* (%)
Etiology			
Iatrogenic	194 (41.8)		
Post-interventional^a^	123 (26.5)		
Drug-related iatrogenia	71 (15.3)		
Traumatic	140 (30.1)		
Decompensated chronic phenomenon	49 (10.5)		
Tumoral	42 (9.0)		
Inflammatory disease	40 (8.6)		
Type of vascular lesion			
Resolved bleeding	154 (33.1)		
Active bleeding	152 (32.7)		
Pseudoaneurysm	66 (14.2)		
Arterial hypertrophy	50 (10.8)		
Diffuse microvascular lesion	23 (4.9)		
Arteriovenous fistula	11 (2.4)		
Aneurysm	9 (1.9)		
Location			
Muscular	110 (23.7)		
Visceral	88 (18.9)		
Splenic	59 (12.7)		
Pelvis	49 (10.5)		
Renal	48 (10.3)		
Bronchial	46 (9.9)		
Hepatic	35 (7.5)		
Urological	10 (2.2)		
Limb	9 (1.9)		
Uterus	7 (1.5)		
Adrenal	3 (0.6)		
Prostate	1 (0.2)		
Artery diameter (mm)		1.8 (1.2–2.6)	7 (1.5)
0.0–0.9	63 (13.8)		
1.0–1.9	195 (42.6)		
2.0–2.9	101 (22.1)		
3.0–3.9	38 (8.3)		
≥4.0	61 (13.3)		

^a^Included complications following surgery, interventional procedures such as biopsies or drainage, or technical medical acts

Initial angiography found predominantly resolved bleeding and active bleeding, accounting for 154 (33.1%) and 152 (32.7%) of 465 arteries respectively. Pseudoaneurysms were found in 14.2% (*n* = 66) of cases, while arterial hypertrophy, diffuse microvascular lesion, arteriovenous fistula, and aneurysm accounted for 10.8%, 4.9%, 2.4%, and 1.9%, respectively. Lesion locations were mostly muscular (23.7%), and visceral (18.9%). All location distributions are detailed in Table 1. Most embolized arteries had a diameter of less than 3 mm (78.5%). Among these, 42.6% measured 1.0–1.9 mm, 22.1% measured 2.0–2.9 mm, and 13.8% measured 0.0–0.9 mm. About a quarter of embolizations were performed over the weekend (i.e., from Saturday 0 A.M. to Sunday 12 P.M.) and/or night shift (from 6 P.M. to 8 A.M.), representing 24.9% and 26.5% respectively.

**Fig. 2:**
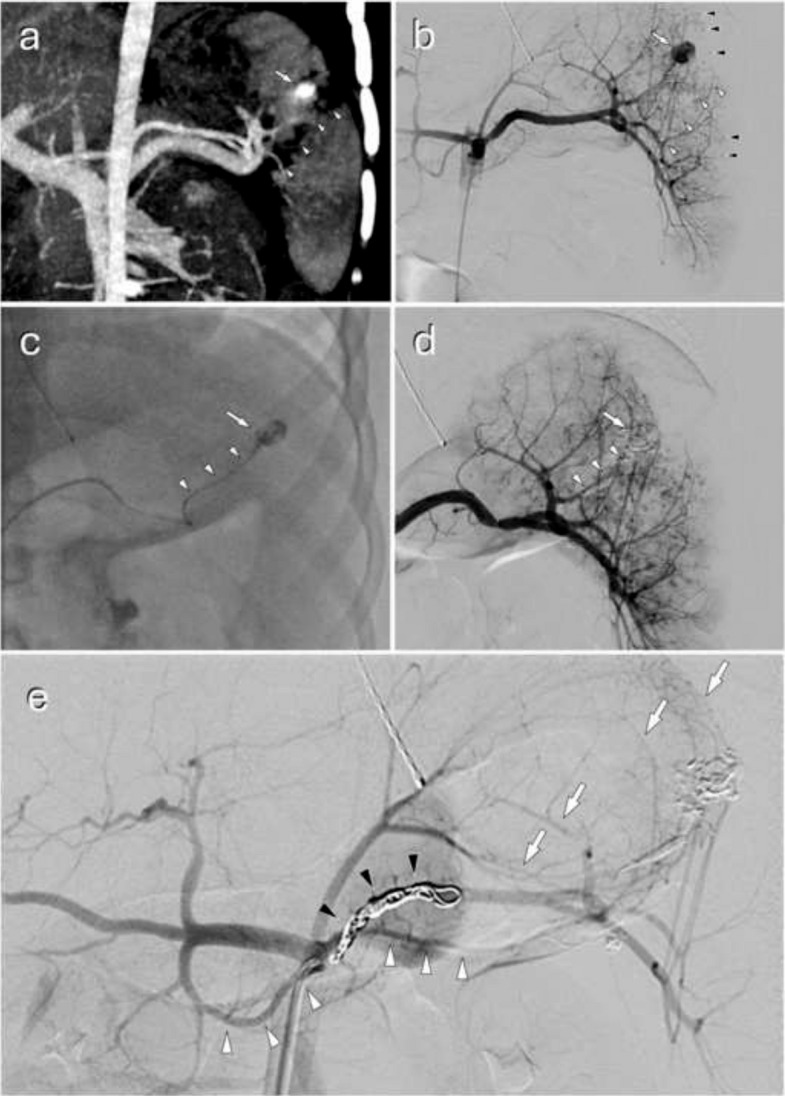
A spleen trauma managed by distal selective and truncal embolization. A 6-year-old patient following a bicycle fall with abdominal trauma. **a **Contrast-enhanced CT revealed a grade IV splenic injury according to the *American Association for the Surgery of Trauma (AAST)* classification, characterized by a 4 cm through-and-through laceration extending to the hilum (arrowheads), associated with hemoperitoneum and an 8 mm splenic pseudoaneurysm (arrow). The patient was hemodynamically stable. After multidisciplinary discussion, a decision was made to proceed with interventional radiology management. **b** Under general anesthesia, a coeliac angiography confirmed CT findings, with 8 mm pseudoaneurysm (arrow), laceration extending to the hilum (white arrowheads), and demonstrated diffuse splenic parenchymal petechiae (black arrowheads). **c** During and **d** after superselective microcatheterization and embolization of both the pseudoaneurysm (arrow) and its feeding branch (arrowheads) using Onyx-18 (Medtronic, Irvine, CA, USA). **e** Truncal embolization was then performed using 7 mm and 4 mm Concerto coils (Medtronic, Irvine, CA, USA) (black arrowheads). Post-embolization angiography demonstrated reperfusion of the splenic parenchyma via collateral flow through the dorsal pancreatic artery (white arrowheads), as well as short gastric vessels (arrows)

**Fig. 3:**
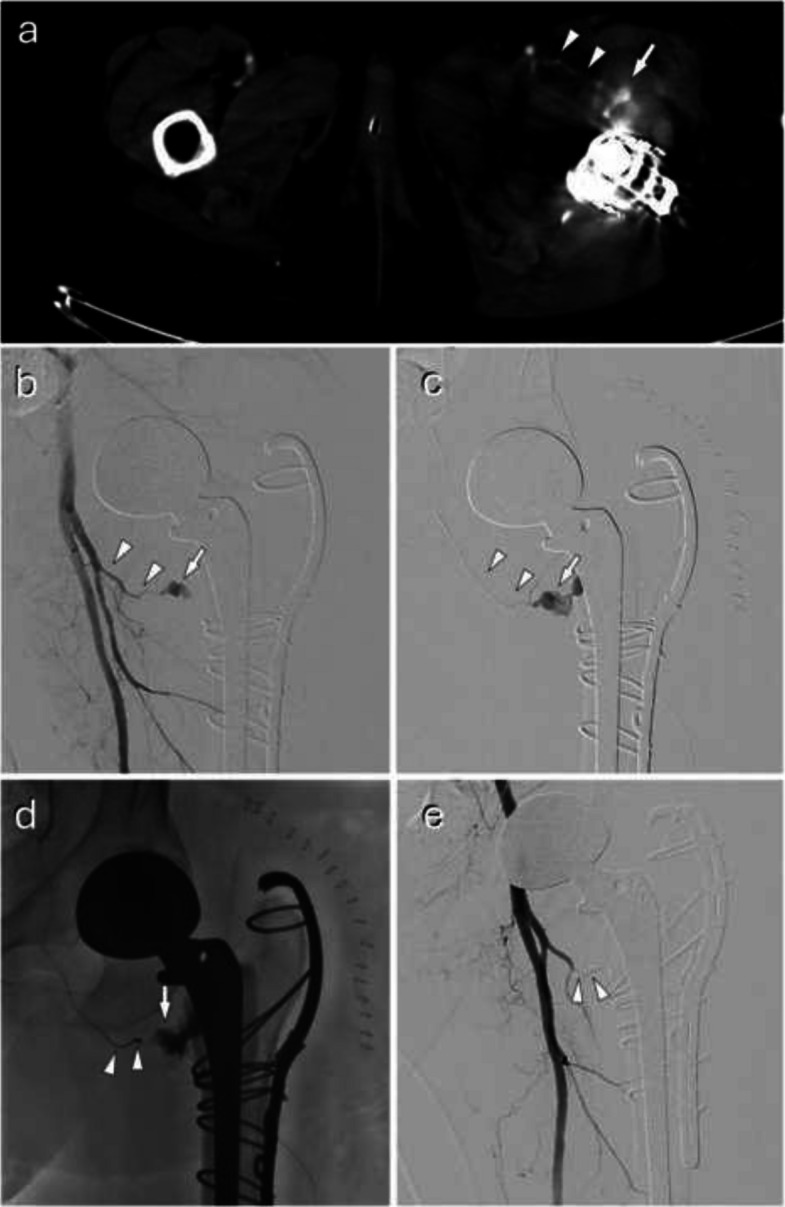
A postoperative active bleeding managed by coiling. A 58-year-old patient undergoing revision of a left total hip arthroplasty with cerclage. Postoperatively, the patient developed a thigh hematoma with acute anemia but remained hemodynamically stable. **a** Contrast-enhanced CT identified active bleeding (arrow) from a proximal branch of the profunda femoris artery: the ascending branch of the lateral circumflex femoral artery (arrowheads). He was managed by interventional radiology after multidisciplinary discussion. The procedure was performed under sedation and local anesthesia. Using a cross-over approach, a 6 F catheter was used for angiography (**b**), and superselective catheterization with a 2.7F microcatheter (**c**), which confirmed CT findings (same annotations). **d** Embolization of the bleeding branch was performed using multiple coils (arrowheads), just proximal to the site of contrast extravasation (arrow). **e** Post-embolization angiography shows complete occlusion of the ascending branch of the lateral circumflex femoral artery by coils (arrowheads), and contrast extravasation resolution

**Fig. 4:**
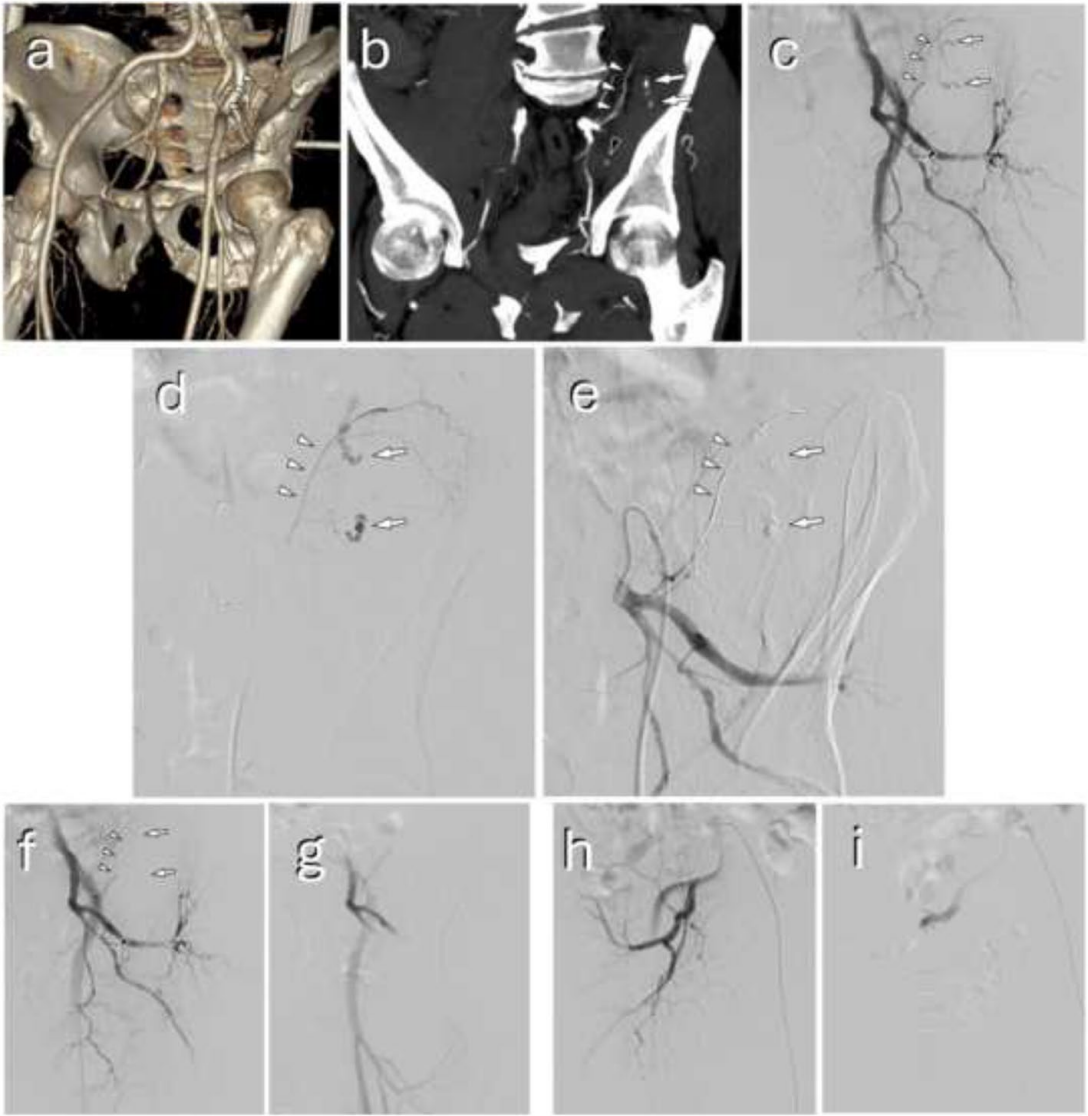
A pelvic trauma managed by selective liquid embolization of active bleeding and bilateral internal iliac resorbable gelatin embolization. A 62-year-old patient presented after a 3-m fall, in a state of hemodynamic instability partially compensated with low-dose vasopressors. **a** Contrast-enhanced CT revealed a Tile C pelvic fracture. A fragment of the left sacral ala was displaced anteriorly (arrowheads), close to the left internal iliac artery, particularly its posterior trunk branches. **b** A hematoma was present in this region, along with a markedly irregular iliolumbar artery (white arrowheads) and active bleeding in its vicinity (arrows). Additional smaller-volume active bleeding was also noted (black arrowhead). Following multidisciplinary discussion, interventional radiology management was initiated. **c** Under local anesthesia, a 6 F ipsilateral catheterization of the left internal iliac artery was performed, and angiography showed two active bleeds (arrows) from the irregular iliolumbar artery (white arrowheads). The pudendal artery appeared clearly dysplastic (black arrowhead), but the active hemorrhage previously visualized in this region had disappeared. **d** Microcatheterization of the iliolumbar artery with a 2.4-F catheter confirmed the source of bleeding, followed by **e** complete Onyx-18 (Medtronic, Irvine, CA, USA) embolization (same annotations). **f** Post-embolization angiography showed complete occlusion of the iliolumbar artery (arrowheads) and contrast extravasation resolution (arrows), with persistent flow through the dysplastic pudendal artery (black arrowhead). **g** This dysplastic vessel was managed with temporary embolization of the internal iliac artery using resorbable gelatin macrofragments. **h** No vascular abnormalities were identified on the right side, yet embolization with resorbable gelatin was also performed (**i**)

Technical success was achieved in 99.1% of cases (Table 2). Some examples of interventions and different embolic agents are presented in Figs. 2, 3, and 4. The 4 (0.9%) cases of failure were all due to catheterization failures. Seven (1.5%) arteries could not be measured: 1 (0.2%) corresponded to a failed catheterization of a thrombosed splenic artery, and the other 6 (1.3%) were cases with no pathological artery found managed by therapeutic abstention. There was a total of 9 (1.9%) cases of abstention, and only 3 (0.6%) cases involved visible and measurable pathological arteries. One (0.2%) complication of asymptomatic arterial dissection occurred in a branch of the superior mesenteric artery during embolization of a centimetric pseudoaneurysm on postoperative day 15 following a cephalic duodenopancreatectomy. There were no off-target embolizations. There were no discrepancies in technical success and complications between reports and the angiography analysis. The agent used was based on three materials in 74.3% of cases: coils (35.7%), liquid agents (22.0%), and resorbable gelatin (16.6%). Combinations of agents were used in 10.8% of cases, almost exclusively to combine coils with another agent (Table 2).

**Table 2:** Procedure and material characteristics

Characteristic	Number (%); *n* = 465
Procedure success	
Yes	461 (99.1)
Catheterization failure	4 (0.9)
Abstention	9 (1.9)
Complication	1 (0.2)
Artery dissection, asymptomatic	1 (0.2)
Off-target embolization	0 (0)
Material	452 (97.2)
Coil	166 (35.7)
Liquid	102 (22.0)
EVOH-based	99 (21.3)
Cyanoacrylate	3 (0.7)
Resorbable gelatin	77 (16.6)
Microparticles	34 (7.3)
Plug	13 (2.8)
Stent	10 (2.2)
Combined	50 (10.8)
Coil + liquid	20 (4.3)
Coil + EVOH-based	16 (3.4)
Coil + cyanoacrylate	4 (0.9)
Coil + resorbable gelatin	17 (3.7)
Coil + microparticles	7 (1.5)
Coil + plug	2 (0.4)
Resorbable gelatin + microparticles	1 (0.2)
Coil + EVOH-based + resorbable gelatin	1 (0.2)
Coil + EVOH-based + microparticles	1 (0.2)
Coil + plug + stent	1 (0.2)

*EVOH* ethylene vinyl alcohol

Regarding distribution of embolic agents according to artery diameter size (Table 3), arteries smaller than 1 mm (13.8% of cases) were treated with liquid agents (i.e., cyanoacrylate or EVOH-based agents) in 46.7% of cases. We note that the use of liquid agents decreased with the vessel diameter. On the other hand, coils were the most widely used embolic agent in all other size ranges, followed by resorbable gelatin for arteries ≥ 2.0 mm.

**Table 3:** Distribution of embolic agents according to artery diameter size

Material	0.0–0.9 mm	1.0–1.9 mm	2.0–2.9 mm	3.0–3.9 mm	≥ 4.0 mm
Coil	21 (33.9)	81 (42.6)	33 (32.7)	12 (31.6)	19 (31.1)
Liquid	29 (46.7)	58 (30.5)	14 (13.9)	1 (2.6)	0 (0)
EVOH-based	27 (43.5)	57 (30.0)	14 (13.9)	1 (2.6)	0 (0)
Cyanoacrylate	2 (3.2)	1 (0.5)	0 (0)	0 (0)	0 (0)
Microparticles	1 (1.6)	9 (4.7)	18 (17.8)	4 (10.5)	2 (3.3)
Plug	0 (0)	0 (0)	0 (0)	3 (7.9)	10 (16.4)
Resorbable gelatin	3 (4.8)	25 (13.2)	23 (22.8)	11 (28.9)	15 (24.6)
Stent	0 (0)	0 (0)	1 (1)	0 (0)	9 (14.8)
Coil + liquid	4 (6.4)	6 (3.2)	3 (3)	4 (10.5)	3 (4.9)
Coil + EVOH-based	3 (4.8)	6 (3.2)	2 (2)	4 (10.5)	1 (1.6)
Coil + cyanoacrylate	1 (1.6)	0 (0)	1 (1)	0 (0)	2 (3.3)
Coil + microparticles	0 (0)	4 (2.1)	2 (2)	1 (2.6)	0 (0)
Coil + plug	0 (0)	0 (0)	0 (0)	1 (2.6)	1 (1.6)
Coil + resorbable gelatin	4 (6.5)	6 (3.2)	5 (5)	1 (2.6)	1 (1.6)
Resorbable gelatin + microparticles	0 (0)	1 (0.5)	0 (0)	0 (0)	0 (0)
Coil + EVOH-based + microparticles	0 (0)	0 (0)	1 (1)	0 (0)	0 (0)
Coil + EVOH-based + resorbable gelatin	0 (0)	0 (0)	1 (1)	0 (0)	0 (0)
Coil + plug + stent	0 (0)	0 (0)	0 (0)	0 (0)	1 (1.6)

Data are numerators used to calculate percentages, and numbers in parentheses are percentages within the column. Percentages are rounded

*EVOH* ethylene vinyl alcohol

Lesion location was sometimes decisive for the type of material used (Table 4). In the pelvis, resorbable gelatin was used in over half (53.1%) of the cases. In the bronchial arteries, 72.7% of embolizations used spherical particles (which were rarely used elsewhere). Plugs were used almost exclusively in truncal embolizations of splenic arteries.

**Table 4:** Distribution of embolic agents according to artery location

Material	Muscular	Visceral	Splenic	Pelvis	Renal	Bronchial	Hepatic	Urological	Limb	Uterus	Adrenal	Prostate
Coil	39 (36.8)	43 (50.6)	34 (58.6)	8 (16.3)	20 (44.4)	3 (6.8)	11 (31.4)	4 (40)	1 (11.1)	0 (0)	3 (100)	0 (0)
Liquid	30 (28.3)	22 (25.9)	7 (12.1)	8 (16.3)	19 (42.2)	3 (6.8)	9 (25.7)	0 (0)	2 (22.2)	1 (14.3)	0 (0)	1 (100)
EVOH-based	29 (27.4)	22 (25.9)	7 (12.1)	8 (16.3)	19 (42.2)	3 (6.8)	7 (20)	0 (0)	2 (22.2)	1 (14.3)	0 (0)	1 (100)
Cyanoacrylate	1 (0.9)	0 (0)	0 (0)	0 (0)	0 (0)	0 (0)	2 (5.7)	0 (0)	0 (0)	0 (0)	0 (0)	0 (0)
Microparticles	0 (0)	0 (0)	0 (0)	0 (0)	0 (0)	32 (72.7)	1 (2.9)	0 (0)	0 (0)	1 (14.3)	0 (0)	0 (0)
Plug	0 (0)	0 (0)	12 (20.7)	0 (0)	1 (2.2)	0 (0)	0 (0)	0 (0)	0 (0)	0 (0)	0 (0)	0 (0)
Resorbable gelatin	25 (23.6)	5 (5.9)	0 (0)	26 (53.1)	0 (0)	2 (4.5)	9 (25.7)	5 (50)	1 (11.1)	4 (57.1)	0 (0)	0 (0)
Stent	1 (0.9)	2 (2.4)	0 (0)	0 (0)	0 (0)	0 (0)	3 (8.6)	0 (0)	4 (44.4)	0 (0)	0 (0)	0 (0)
Coil + liquid	4 (3.8)	9 (10.6)	2 (3.4)	1 (2)	1 (2.2)	0 (0)	2 (5.8)	0 (0)	1 (11.1)	0 (0)	0 (0)	0 (0)
Coil + EVOH-based	4 (3.8)	8 (9.4)	1 (1.7)	1 (2)	1 (2.2)	0 (0)	1 (2.9)	0 (0)	0 (0)	0 (0)	0 (0)	0 (0)
Coil + cyanoacrylate	0 (0)	1 (1.2)	1 (1.7)	0 (0)	0 (0)	0 (0)	1 (2.9)	0 (0)	1 (11.1)	0 (0)	0 (0)	0 (0)
Coil + microparticles	0 (0)	0 (0)	0 (0)	0 (0)	3 (6.7)	4 (9.1)	0 (0)	0 (0)	0 (0)	0 (0)	0 (0)	0 (0)
Coil + plug	0 (0)	1 (1.2)	1 (1.7)	0 (0)	0 (0)	0 (0)	0 (0)	0 (0)	0 (0)	0 (0)	0 (0)	0 (0)
Coil + resorbable gelatin	7 (6.6)	3 (3.5)	2 (3.4)	5 (10.2)	0 (0)	0 (0)	0 (0)	0 (0)	0 (0)	0 (0)	0 (0)	0 (0)
Resorbable gelatin + microparticles	0 (0)	0 (0)	0 (0)	0 (0)	0 (0)	0 (0)	0 (0)	0 (0)	0 (0)	1 (14.3)	0 (0)	0 (0)
Coil + EVOH-based + resorbable gelatin	0 (0)	0 (0)	0 (0)	1 (2)	0 (0)	0 (0)	0 (0)	0 (0)	0 (0)	0 (0)	0 (0)	0 (0)
Coil + EVOH-based + microparticles	0 (0)	0 (0)	0 (0)	0 (0)	1 (2.2)	0 (0)	0 (0)	0 (0)	0 (0)	0 (0)	0 (0)	0 (0)
Coil + plug + stent	0 (0)	0 (0)	0 (0)	0 (0)	0 (0)	0 (0)	0 (0)	1 (10)	0 (0)	0 (0)	0 (0)	0 (0)

Data are numerators used to calculate percentages, and numbers in parentheses are percentages within the column. Percentages are rounded

*EVOH* ethylene vinyl alcohol

For active bleeding, liquid agents (38.6%) and coils (37.3%) were the most commonly used (Table 5). In cases of spontaneously resolved bleeding (33.1% of cases), resorbable gelatin was most used (40.4%). For pseudoaneurysms and arteriovenous fistula (14.2% and 2.4% of cases, respectively), coils were widely used (50.8% and 72.7%, respectively).

**Table 5:** Distribution of embolic agents according to vascular lesion type

Material	Aneurysm	Pseudoaneurysm	Active bleeding	Resolved bleeding	Arterial hypertrophy	Arteriovenous fistula	Diffuse microvascular lesion
Coil	6 (66.7)	33 (50.8)	56 (37.3)	51 (34.9)	2 (4.2)	8 (72.7)	10 (43.5)
Liquid	2 (22.2)	16 (24.6)	58 (38.6)	21 (14.4)	4 (8.3)	1 (9.1)	0 (0)
EVOH-based	2 (22.2)	15 (23.1)	56 (37.3)	21 (14.4)	4 (8.3)	1 (9.1)	0 (0)
Cyanoacrylate	0 (0)	1 (1.5)	2 (1.3)	0 (0)	0 (0)	0 (0)	0 (0)
Microparticles	0 (0)	0 (0)	0 (0)	1 (0.7)	33 (68.8)	0 (0)	0 (0)
Plug	0 (0)	0 (0)	2 (1.3)	0 (0)	0 (0)	0 (0)	11 (47.8)
Resorbable gelatin	0 (0)	2 (3.1)	11 (7.3)	59 (40.4)	5 (10.4)	0 (0)	0 (0)
Stent	0 (0)	3 (4.6)	4 (2.7)	3 (2.1)	0 (0)	0 (0)	0 (0)
Coil + liquid	1 (11.1)	6 (9.2)	9 (6.0)	3 (2.1)	0 (0)	1 (9.1)	0 (0)
Coil + EVOH-based	1 (11.1)	5 (7.7)	7 (4.7)	2 (1.4)	0 (0)	1 (9.1)	0 (0)
Coil + Cyanoacrylate	0 (0)	1 (1.5)	2 (1.3)	1 (0.7)	0 (0)	0 (0)	0 (0)
Coil + microparticles	0 (0)	3 (4.6)	0 (0)	0 (0)	3 (6.3)	1 (9.1)	0 (0)
Coil + plug	0 (0)	0 (0)	1 (0.7)	0 (0)	0 (0)	0 (0)	1 (4.3)
Coil + resorbable gelatin	0 (0)	1 (1.5)	7 (4.7)	8 (5.5)	0 (0)	0 (0)	1 (4.3)
Resorbable gelatin + microparticles	0 (0)	0 (0)	0 (0)	0 (0)	1 (2.1)	0 (0)	0 (0)
Coil + EVOH-based + resorbable gelatin	0 (0)	0 (0)	1 (0.7)	0 (0)	0 (0)	0 (0)	0 (0)
Coil + EVOH-based + microparticles	0 (0)	1 (1.5)	0 (0)	0 (0)	0 (0)	0 (0)	0 (0)
Coil + plug + stent	0 (0)	0 (0)	1 (0.7)	0 (0)	0 (0)	0 (0)	0 (0)

Data are numerators used to calculate percentages, and numbers in parentheses are percentages. Percentages are rounded

*EVOH* ethylene vinyl alcohol

Although this depends on the country of application, the cost-of-use was highest for the coiling embolization strategy, while cases using liquid agents were 2.5 to 6 times less expensive, as shown in Table 6. Full details of the material used are given in the Supplementary material Table S1.

**Table 6:** Cost evaluation of embolic materials

Material	Embolizations using it	Number by embolization*	Unit cost (France, €)*	Cost by embolization (France, €)* ▽
Global	452 (100)	2.8 (3.1)	358.8 (213.0)	1023.3 (1368.2); 581.4 [135.6–1182.7]**
Coil	215 (47.6)	4.5 (3.7)	357.8 (167.9)	1685.1 (1650.4)
Stent	11 (2.4)	1.3 (0.6)	792.1 (407.3)	930.6 (506.8)
EVOH-based	117 (25.9)	1.1 (0.4)	581.4 (0.0)	631.1 (223.5)
Plug	16 (3.5)	1 (0.0)	348.4 (0.0)	348.4 (0)
Cyanoacrylate	7 (1.5)	1 (0)	274.0 (0.0)	274.0 (0)
Microparticles	43 (9.5)	1.4 (0.9)	117.9 (0.0)	167.3 (100.4)
Resorbable gelatin	96 (21.2)	1.0 (0.2)	40.0 (0.0)	41.7 (8.0)

Data are numerators used to calculate percentages, and numbers in parentheses are percentages, except where otherwise indicated. Percentages are rounded

*EVOH* ethylene vinyl alcohol

^*^Result given in the form: Mean (standard deviation)

^*^*Result given in the form: Median [Q1–Q3]

▽Materials are classified according to this category, in descending order

## Discussion

This large cohort shows the high proportion of emergency embolization procedures for iatrogenic indications (41.8%), either post-interventional or antithrombotic-related. This iatrogenic proportion is consistent with a 2018 study where post-interventional represented 38.8% and anticoagulation 8.0% of indications [5]. This clearly underlines the role of interventional radiology as a major aid in the management of complications. Traumatic contexts were also widely represented (30.1%), whereas oncological contexts accounted for only 9% of cases. The high technical success rate of 99.1% underscores the effectiveness of these types of embolization, with only one (0.2%) asymptomatic per-procedural complication. The size of the arteries involved was less than 1.9 mm in more than 50% of cases, which is not very intuitive. In these situations, it's often easier to inject a small volume of liquid agent than a solid one [14]. Firstly, because they are not always available in such small diameters, especially if < 1 mm. Secondly, because they will have an increased length once deployed in the artery. This final occlusion length is difficult to assess before the material is released. Finally, resolution of arterial spasm following treatment of the bleeding may result in undersized non-liquid materials relative to the real diameter of the affected artery. This is why in our team we use mainly liquid agents, especially in cases of active bleeding. This study highlights the high use of liquid EVOH-based agents in comparison with cyanoacrylate adhesives (21.3 and 0.7%, respectively). The difference found here may be explained by a center effect, and that is why we decided to describe results by grouping them under the term “liquid agent”. Some articles in the literature give information on their use in the context of emergency embolization, sometimes focusing on EVOH-based agents, described as safe and effective [15–17]. In an article about musculoskeletal tumor embolization, the use of EVOH-based agents and cyanoacrylate adhesives was close (19% and 16%, respectively) [18]. Interestingly, we demonstrate that for the economic aspect in our country, using coils, which very often required several coils, was more expensive than using liquid agents. In emergency settings, awareness of cost differences between embolic agents may help optimize resource allocation and planning, while maintaining high procedural efficacy.

Active bleeding and bleeding that had spontaneously resolved during the care process, each account for about a third of cases, while pseudoaneurysms or arteriovenous fistulas represented 14.2% and 2.4%, respectively [2].

Our study has several limitations. Firstly, this is a retrospective study that only focuses on per-procedure data and does not consider post-operative clinical outcomes. Secondly, the monocentric character of our study implies that the results are not necessarily generalizable, especially as the use of embolization equipment is highly dependent on the operator’s habits. However, it is based on a large cohort of patients involving 15 different operators, reinforcing the relevance and potential generalizability of the results. In our center, there are no predefined or standardized criteria used by operators for embolic agent selection in emergency settings. This absence of strict selection rules is precisely what motivated the study to document real-life operator habits and analyze actual patterns of practice emerging from routine clinical activity, by systematically collecting embolic materials used. The absence of detailed clinical follow-up and clinical outcome data is a limitation of our study, but our work was designed primarily as a technical overview. Finally, only patients classified as emergencies were included, meaning that it was not possible to study relative emergency cases, where patients were previously hospitalized from a few hours to a few days. Nevertheless, in our experience, these patients appear to be managed in a similar way to those studied here. Neurological and cervico-facial emergency embolizations were not included because these procedures are performed in our institution by a dedicated team of interventional neuroradiologists. Similarly, obstetrical embolizations were not included because obstetric emergencies are managed in a separate specialized center located adjacent to our maternity unit where all obstetric cases are systematically referred.

## Conclusions

This large cohort confirmed that, in emergency embolization local practice, small-caliber vessels (1.0–1.9 mm) were the most frequently targeted. In cases of active bleeding, liquid embolic agents were widely favored; their cost was lower than that of coils in our country's health care economic system, and the overall technical success rate was very high. Moreover, a substantial proportion of interventions were performed on lesions identified by CT scan, although the bleeding had already stopped spontaneously.

## Supplementary Information

Supplementary Material 1: Table S1. Detailed characteristics of embolic agents.

## Data Availability

The datasets generated during and/or analyzed during the current study are available from the corresponding author on reasonable request.

## Abbreviation

EVOH: Ethylene vinyl alcohol
