# Supplementary material for: Embolic agents in emergency care: a large retrospective cohort study
Source: CVIR Endovasc. 2026 Feb 3;9:13. doi: 10.1186/s42155-026-00651-5 (PMC12868507; doi:10.1186/s42155-026-00651-5)
Supplement: Supplementary file 1 — Supplementary Material 1: Table S1. Detailed characteristics of embolic agents. [file 42155_2026_651_MOESM1_ESM.docx]

**Supplementary Material Table 1.** Detailed characteristics of embolic agents

| Embolization Material | Number (%); n=1286 | Number (% Per Category) | Embolization Material (continued) | Number (%); n=1286 | Number (% Per Category) |
| --- | --- | --- | --- | --- | --- |
| Plug | 16 (1.2) |  | Coil (continued) |  |  |
| Amplatzer Vasc. Plug 9x8 | 4 (0.3) | 4 (25) | Interlock 2x4 | 1 (0.1) | 1 (0.1) |
| Amplatzer Vasc. Plug 9x10 | 2 (0.2) | 2 (12.5) | Interlock 2x6 | 3 (0.2) | 3 (0.3) |
| Amplatzer Vasc. Plug A 5 | 1 (0.1) | 1 (6.3) | Interlock 3x6 | 4 (0.3) | 4 (0.4) |
| Amplatzer Vasc. Plug II | 1 (0.1) | 1 (6.3) | Interlock 4x8 | 5 (0.4) | 5 (0.5) |
| Amplatzer Vasc. Plug 4 | 6 (0.5) | 6 (37.5) | Interlock 5x8 | 1 (0.1) | 1 (0.1) |
| Amplatzer II 8-4 | 2 (0.2) | 2 (12.5) | Interlock 6x10 | 3 (0.2) | 3 (0.3) |
| Coil | 961 (74.7) |  | Interlock 8x20 | 1 (0.1) | 1 (0.1) |
| Axium Prime 3d 1x3 | 2 (0.2) | 2 (0.2) | Interlock 12x20 | 1 (0.1) | 1 (0.1) |
| Axium Prime 3d 1.5x3 | 2 (0.2) | 2 (0.2) | Interlock 14x30 | 1 (0.1) | 1 (0.1) |
| Axium Prime 2x1 | 1 (0.1) | 1 (0.1) | Nester 3x2 | 51 (4) | 51 (5.3) |
| Axium Prime 2x3 | 1 (0.1) | 1 (0.1) | Nester 3x3 | 7 (0.5) | 7 (0.7) |
| Axium Prime 2x6 | 2 (0.2) | 2 (0.2) | Nester 3x5 | 43 (3.3) | 43 (4.5) |
| Axium Prime 2x8 | 3 (0.2) | 3 (0.3) | Nester 3x14 | 20 (1.6) | 20 (2.1) |
| Axium Prime 4x8 | 1 (0.1) | 1 (0.1) | Nester 4x7 | 8 (0.6) | 8 (0.8) |
| Azur 2x4 | 2 (0.2) | 2 (0.2) | Nester 4x14 | 13 (1) | 13 (1.4) |
| Azur 2x4 Hydrocoil | 3 (0.2) | 3 (0.3) | Nester 5x3 | 3 (0.2) | 3 (0.3) |
| Azur 3x2 | 1 (0.1) | 1 (0.1) | Nester 5x14 | 6 (0.5) | 6 (0.6) |
| Azur 3x8 | 1 (0.1) | 1 (0.1) | Nester 6x14 | 7 (0.5) | 7 (0.7) |
| Azur 4x5 Hydrocoil | 2 (0.2) | 2 (0.2) | Nester 7x2 | 44 (3.4) | 44 (4.6) |
| Azur 4x15 Hydrocoil | 4 (0.3) | 4 (0.4) | Nester 7x3 | 49 (3.8) | 49 (5.1) |
| Azur 5x15 | 1 (0.1) | 1 (0.1) | Nester 8x14 | 4 (0.3) | 4 (0.4) |
| Azur 5x16 | 1 (0.1) | 1 (0.1) | Nester 10x14 | 5 (0.4) | 5 (0.5) |
| Azur 6x10 Hydrocoil | 1 (0.1) | 1 (0.1) | Nester 14x10 | 3 (0.2) | 3 (0.3) |
| Azur 6x20 | 1 (0.1) | 1 (0.1) | Pod Packing 15 | 7 (0.5) | 7 (0.7) |
| Azur 6x20 Hydrocoil | 1 (0.1) | 1 (0.1) | Pod 4 - 30 | 3 (0.2) | 3 (0.3) |
| Azur 7x24 | 3 (0.2) | 3 (0.3) | Pod 5 Packing 30 | 5 (0.4) | 5 (0.5) |
| Azur 18-8x20 Hydrocoil | 1 (0.1) | 1 (0.1) | Packing Coil 30 | 5 (0.4) | 5 (0.5) |
| Azur 9x28 | 3 (0.2) | 3 (0.3) | Pod Packing 45 | 5 (0.4) | 5 (0.5) |
| Azur 18-10x20 Hydrocoil | 2 (0.2) | 2 (0.2) | Pod 6 Paking 50 | 10 (0.8) | 10 (1) |
| Azur 10x32 | 1 (0.1) | 1 (0.1) | Pod 8 - 60 | 4 (0.3) | 4 (0.4) |
| Azur 12x20 Hydrocoil | 1 (0.1) | 1 (0.1) | Pod 10 - 60 | 1 (0.1) | 1 (0.1) |
| Azur 12x38 | 1 (0.1) | 1 (0.1) | Ruby 3x5 | 3 (0.2) | 3 (0.3) |
| Azur 15x30 | 1 (0.1) | 1 (0.1) | Ruby 4x6 | 5 (0.4) | 5 (0.5) |
| Azur 15x30 Hydrocoil | 1 (0.1) | 1 (0.1) | Ruby 4x15 | 4 (0.3) | 4 (0.4) |
| Azur 20x30 Cx | 1 (0.1) | 1 (0.1) | Ruby 6x20 | 6 (0.5) | 6 (0.6) |
| Azur 20x30 Hydro | 2 (0.2) | 2 (0.2) | Ruby 6x30 | 8 (0.6) | 8 (0.8) |
| Azur 20x40 | 1 (0.1) | 1 (0.1) | Ruby 6x35 | 1 (0.1) | 1 (0.1) |
| Concerto 1x2 | 1 (0.1) | 1 (0.1) | Ruby 8x25 | 7 (0.5) | 7 (0.7) |
| Concerto 1.5x4 | 1 (0.1) | 1 (0.1) | Ruby 8x35 | 4 (0.3) | 4 (0.4) |
| Concerto 2x4 | 83 (6.5) | 83 (8.6) | Ruby 8x40 | 3 (0.2) | 3 (0.3) |
| Concerto 2x4 3d | 3 (0.2) | 3 (0.3) | Ruby 8x60 | 1 (0.1) | 1 (0.1) |
| Concerto 2x6 | 5 (0.4) | 5 (0.5) | Ruby 10x35 | 3 (0.2) | 3 (0.3) |
| Concerto 2x6 3d | 4 (0.3) | 4 (0.4) | Ruby 14x60 | 2 (0.2) | 2 (0.2) |
| Concerto 2x8 | 27 (2.1) | 27 (2.8) | Ruby 18x60 | 1 (0.1) | 1 (0.1) |
| Concerto Helix 3x4 | 53 (4.1) | 53 (5.5) | Ruby 20x60 | 5 (0.4) | 5 (0.5) |
| Concerto 3d 3x4 | 11 (0.9) | 11 (1.1) | Ruby 24x60 | 3 (0.2) | 3 (0.3) |
| Concerto 3x6 | 19 (1.5) | 19 (2) | Ruby 28x60 | 3 (0.2) | 3 (0.3) |
| Concerto 3x6 3d | 8 (0.6) | 8 (0.8) | Ruby 32x60 | 2 (0.2) | 2 (0.2) |
| Concerto 3x8 | 10 (0.8) | 10 (1) | Ruby 36x60 | 2 (0.2) | 2 (0.2) |
| Concerto 3x8 3d | 2 (0.2) | 2 (0.2) | Spifu microcoil 2,5x5p10 | 2 (0.2) | 2 (0.2) |
| Concerto 4x10 | 86 (6.7) | 86 (8.9) | EVOH-based | 127 (9.9) |  |
| Concerto 4x8 | 18 (1.4) | 18 (1.9) | Onyx18 (bottle) | 66 (5.1) | 66 (52) |
| Concerto 4x8 3d | 5 (0.4) | 5 (0.5) | Onyx34 | 55 (4.3) | 55 (43.3) |
| Concerto 4x20 | 1 (0.1) | 1 (0.1) | Squid 18 | 6 (0.5) | 6 (4.7) |
| Concerto 5x15 | 25 (1.9) | 25 (2.6) | Cyanoacrylate | 7 (0.5) |  |
| Concerto 5x20 | 32 (2.5) | 32 (3.3) | Histioacryl-Lipiodol | 3 (0.2) | 3 (42.9) |
| Concerto 6x20 | 12 (0.9) | 12 (1.2) | Glubran-Lipiodol | 4 (0.3) | 4 (57.1) |
| Concerto 6x20 3d | 4 (0.3) | 4 (0.4) | Resorbable Gelatin | 100 (7.8) |  |
| Concerto 7x30 | 11 (0.9) | 11 (1.1) | Gelfoam | 79 (6.1) | 79 (79) |
| Concerto 8x30 | 8 (0.6) | 8 (0.8) | Embocube 2,5x50 | 15 (1.2) | 15 (15) |
| Concerto 10x30 | 4 (0.3) | 4 (0.4) | Embocube5x50 | 6 (0.5) | 6 (6) |
| Concerto 16x40 | 1 (0.1) | 1 (0.1) | Microparticles | 61 (4.7) |  |
| Flow Coils Spifu 2,5x10 | 2 (0.2) | 2 (0.2) | Embozen 700 | 27 (2.1) | 27 (44.3) |
| Flow Coils Spifu 2,5x20 | 3 (0.2) | 3 (0.3) | Embozen 900 | 21 (1.6) | 21 (34.4) |
| Idc 2x4 | 16 (1.2) | 16 (1.7) | Embozen 1100 | 2 (0.2) | 2 (3.3) |
| Idc 3x6 | 16 (1.2) | 16 (1.7) | Embogold 500-700 | 3 (0.2) | 3 (4.9) |
| Idc 3x10 | 15 (1.2) | 15 (1.6) | Embogold 700-900 | 8 (0.6) | 8 (13.1) |
| Idc 4x12 | 13 (1) | 13 (1.4) | Stents | 14 (1.1) |  |
| Idc 5x15 | 6 (0.5) | 6 (0.6) | Bentley Begraft 6x38 | 2 (0.2) | 2 (14.3) |
| Idc 6x10 | 4 (0.3) | 4 (0.4) | Bentley Begraft 7x23 | 1 (0.1) | 1 (7.1) |
| Idc 6x20 | 5 (0.4) | 5 (0.5) | Bentley Begraft 7x27 | 3 (0.2) | 3 (21.4) |
| Idc 7x20 | 1 (0.1) | 1 (0.1) | Bentley Begraft 8x27 | 3 (0.2) | 3 (21.4) |
| Idc 8x20 | 3 (0.2) | 3 (0.3) | Bentley Begraft 8x37 | 2 (0.2) | 2 (14.3) |
| Idc 10x20 | 2 (0.2) | 2 (0.2) | Bentley Begraft 9x37 | 1 (0.1) | 1 (7.1) |
| Idc 12x20 | 1 (0.1) | 1 (0.1) | Viabann 6x2,5 | 1 (0.1) | 1 (7.1) |
| Interlock 2x3 | 4 (0.3) | 4 (0.4) | Fluency Plus 12x5 | 1 (0.1) | 1 (7.1) |

Note.—Data are numerators used to calculate percentages, and numbers in parentheses are percentages. Percentages are rounded.

EVOH: Ethylene vinyl alcohol

*Amplatzer Vascular Plug (Abbott, Plymouth, MN, USA), Interlock (Medtronic, Irvine, CA, USA), Concerto (Medtronic, Irvine, CA, USA), Axium Prime (Medtronic, Irvine, CA, USA), Nester (Cook Medical, Bloomington, IN, USA), Azur (Terumo, Tokyo, Japan), Azur Hydrocoil (Terumo, Tokyo, Japan), Ruby (Penumbra, Alameda, CA, USA), POD (Penumbra, Alameda, CA, USA), Packing Coil (Penumbra, Alameda, CA, USA), Spifu microcoil (Balt, Montmorency, France), Flow Coils Spifu (Balt, Montmorency, France), IDC (Boston Scientific, Marlborough, MA, USA), Onyx 18 (Medtronic, Irvine, CA, USA), Onyx 34 (Medtronic, Irvine, CA, USA), Squid 18 (Balt, Montmorency, France), Histoacryl (B. Braun, Melsungen, Germany), Glubran (GEM S.r.l., Viareggio, Italy), Lipiodol (Guerbet, Villepinte, France), Gelfoam (Pfizer, New York, NY, USA), Embocube (Terumo, Tokyo, Japan), Embozen (Boston Scientific, Marlborough, MA, USA), Embogold (Boston Scientific, Marlborough, MA, USA), Bentley BeGraft (Bentley InnoMed, Hechingen, Germany), Viabahn (W. L. Gore & Associates, Flagstaff, AZ, USA), Fluency Plus (Bard Peripheral Vascular, Tempe, AZ, USA)*
